# Urine β2-Microglobulin and Retinol-Binding Protein and Renal Disease Progression in IgA Nephropathy

**DOI:** 10.3389/fmed.2021.792782

**Published:** 2021-12-22

**Authors:** Xiaoqi Shen, Jun Cheng, Guizhen Yu, Xiayu Li, Heng Li, Jianghua Chen

**Affiliations:** ^1^Kidney Disease Center, The First Affiliated Hospital, Zhejiang University School of Medicine, Hangzhou, China; ^2^Key Laboratory of Kidney Disease Prevention and Control Technology, Hangzhou, China; ^3^Institute of Nephrology, Zhejiang University, Hangzhou, China

**Keywords:** IgA nephropathy, interstitial fibrosis, tubular atrophy, urine β2-microglobulin, urine retinol binding protein

## Abstract

**Background:** Tubulointerstitial involvement has been reported to have a decisive influence on the progression of IgA nephropathy (IgAN). High levels of urine β2-microglobulin (β2-MG) and retinol-binding protein (RBP) were observed in patients with IgAN with tubulointerstitial lesions. However, their roles in disease progression remain unclear. This study aimed to evaluate the associations of urine β2-MG and RBP with the progression of IgAN.

**Methods:** We retrospectively investigated a cohort of 2,153 patients with IgAN. Clinical and pathological features, outcomes, and urine β2-MG, and RBP at the time of biopsy were collected. The associations, of urine β2-MG and RBP with the composite renal outcome, defined as a decline in estimated glomerular filtration rate (eGFR) of ≥50% from baseline or end-stage renal disease (ESRD), were examined using restricted cubic splines and the Cox proportional hazards models.

**Results:** During a median follow-up of 20.40 months, 140 (6.50%) patients reached the composite renal outcomes. Restricted cubic splines showed that patients with higher urinary β2-MG and RBP levels had worse renal outcomes. The Cox regression analysis revealed that urine β2-MG and RBP were associated with a risk of the composite renal outcome in the multivariate adjusted model [+1 SD for log β2-MG, hazard ratio (HR) = 1.462, 95% CI: 1.136–1.882, *p* = 0.003; +1 SD for log RBP, HR = 1.972, 95% CI: 1.486–2.617, *p* = 0.001]. The associations were detectable within patients with baseline eGFR <90 ml/min/1.73 m^2^ (+1 SD for log β2-MG, HR = 1.657, 95% CI: 1.260–2.180, *p* < 0.001; +1 SD for log RBP, HR = 1.618, 95% CI: 1.199–2.183, *p* = 0.002), but not among patients with eGFR ≥90 ml/min/1.73 m^2^.

**Conclusion:** Higher levels of urine β2-MG and RBP were independent risk factors for renal disease progression in IgAN.

## Introduction

IgA nephropathy (IgAN) is the most prevalent type of primary glomerulonephritis globally, defined as prominent IgA deposition in the glomerular mesangial area (1, 2). Nearly 20–30% of patients with IgAN develop end-stage renal disease (ESRD) eventually and need renal replacement therapies (3, 4). Persistent proteinuria, hypertension, impaired renal function, and the Oxford classification of MEST-C scores are all the established risk factors for poor renal outcomes (5–11). Previous studies indicated that tubulointerstitial injury was a major contributor to the loss of renal function even in primary glomerular diseases (12, 13). Interstitial fibrosis (IF) and tubular atrophy (TA) are common pathological changes and predict renal progression in IgAN (14, 15). Renal pathology is the gold standard for the evaluation of tubulointerstitial lesions. However, the biopsy is an invasive procedure and cannot be used as a routine method. Meanwhile, due to the variability of clinical manifestations of IgAN, patients in the absence of apparent symptoms may never be aware or undergo a renal biopsy. Therefore, it is clinically valuable to find non-invasive biomarkers that predict tubulointerstitial lesions and renal disease progression. Both the β2-microglobulin (β2-MG) and retinol-binding protein (RBP) are low-molecular-weight proteins (11.8 and 21 kD, respectively) that are present in low concentrations in the plasma of healthy people and are freely filtered by the normal glomerulus and then almost completely reabsorbed and catabolized by cells of the proximal tubules (16, 17). Increased levels of urine β2-MG and RBP were found in patients with renal tubulointerstitial damage (18–20). However, their roles in the long-term outcome of IgAN have not been well-assessed. In this study, we aimed to identify the effects of baseline urinary β2-MG and RBP levels on renal disease progression in patients with IgAN.

## Materials and Methods

### Study Population

In this single-center retrospective cohort study, we collected clinical and pathological data from patients with newly diagnosed IgAN between January 2002 and December 2019 in the First Affiliated Hospital, Zhejiang University School of Medicine. Patients were diagnosed based on the dominant deposition of IgA in the mesangial area by immunofluorescence. The exclusion criteria were as follows: (1) missing urinary β2-MG and RBP data at the time of renal biopsy; (2) patients with IgA vasculitis, systemic lupus erythematosus, hepatitis B virus infection, other secondary IgAN, and acute kidney injury; (3) patients whose baseline estimated glomerular filtration rate (eGFR) was <15 ml/min/1.73 m^2^; and (4) follow-up for <3 months. All the patients included in this study had provided their written informed consent for the renal biopsy. The study protocols conformed to the provisions of the Declaration of Helsinki and were approved by the Ethics Committee (IIT20210679A).

### Data Collection and Definition

Clinical data, including sex, age, systolic/diastolic blood pressure, serum creatinine (SCr), 24-h urine protein excretion, urinary β2-MG (milligrams per mol creatinine), and urinary RBP (milligrams per mol creatinine) levels at the time of renal biopsy, were collected. Levels of urine β2-MG and RBP were measured in spot urine samples. We used urine protein creatinine ratio (UPCR) if urinary protein concentration in the 24-h urine samples was not available. Histopathologic elements were evaluated according to the Oxford classification (14). The eGFR was calculated using the Chronic Kidney Disease Epidemiology Collaboration (CKD-EPI) equation (21). ESRD was defined as the initiation of renal replacement therapies including hemodialysis, peritoneal dialysis, renal transplantation, or an eGFR <15 ml/min/1.73 m^2^. The composite renal outcome was defined as a decline in eGFR of ≥50% from baseline or ESRD. For the analysis of the associations of urine β2-MG and RBP with the composite renal outcome, patients were divided into 3 groups according to the tertiles of the baseline urine β2-MG and RBP.

### Statistical Analyses

Continuous variables are presented as mean ± SD or median and interquartile range (IQR), while categorical variables are expressed as a number or percentage. Skewed variables (urine β2-MG, proteinuria, and urine RBP) underwent a logarithmic transformation to improve normality before analysis. Categorical variables were analyzed with the chi-squared test, whereas continuous variables were compared using the Student's *t*-test, the Mann–Whitney *U*-test, the Kruskal–Wallis *H*-test, or the ANOVA, as appropriate. Correlations of baseline urine β2-MG, urine RBP with eGFR, and proteinuria were evaluated by Pearson's correlation analysis, while correlations of urine β2-MG and RBP with the Oxford classification of the MEST-C scores were evaluated by Spearman's correlation analysis. To determine the associations of urinary β2-MG and RBP levels with renal disease progression, restricted cubic splines curves based on the multivariate adjusted Cox models were used. Cumulative renal survival was estimated by the Kaplan–Meier method. Furthermore, the Cox proportional hazards models were used to evaluate the associations of urine β2-MG and urine RBP with the risk of renal disease progression events. Traditional risk factors including age, sex, mean arterial pressure (MAP), proteinuria, SCr, and the MEST-C scores were adjusted in the multivariate adjusted Cox models. The analyses were performed by the SPSS 18.0 software (SPSS Incorporation, Chicago, Illinois, USA) and the Stata/SE 15.1 software (StataCorp LP., Texas, USA). *p*-value of <0.05 was considered statistically significant.

## Results

**Figure 1:**
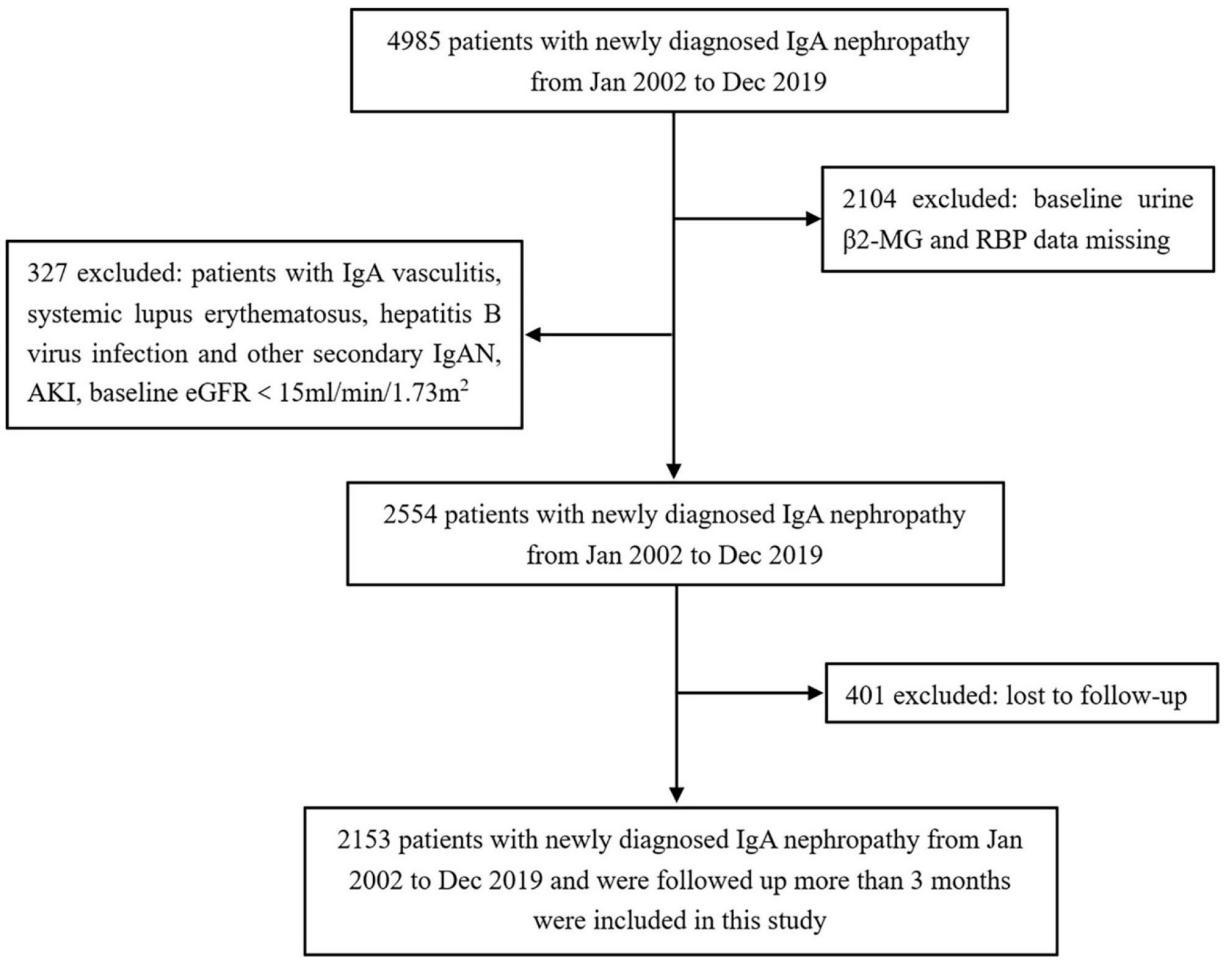
Flowchart of participants in the cohort. β2-MG, β2-microglobulin; RBP, retinol-binding protein; IgAN, IgA nephropathy; AKI, acute kidney injury; eGFR, estimated glomerular filtration rate.

Of 4,985 patients screened, 2,153 patients with IgAN met the selection criteria and were included in this study (Figure 1). The baseline clinical and pathological characteristics of the study population are shown in Table 1. There were 1,022 (47.47%) men and the mean age at the time of renal biopsy was 38.91 ± 12.47 years. The baseline urinary β2-MG and RBP levels were 56.0 mg/mol Cr (IQR, 30.0–127.5) and 783.0 mg/mol Cr (IQR, 267.0–1774.5), respectively. On biopsy, the 24-h proteinuria level was 0.87 g/day (IQR, 0.45–1.69) and the average eGFR was 82.93 ± 29.28 ml/min/1.73 m^2^. In 1,741 patients whose renal biopsies were scored by the Oxford classification, the distributions of M1, E1, S1, T1-T2, and C1-C2 were 11.32, 5.69, 70.13, 11.60, and 40.44%, respectively. After a median follow-up of 20.40 months (IQR, 9.13–43.60), the composite renal outcomes occurred in 140 (6.50%) patients including 83 (3.86%) patients with ESRD.

**Table 1:** Clinical and pathological characteristics of patients with IgA nephropathy.

**Characteristic**	**Value**, ***n*** **= 2,153**
**Baseline**	
Male sex, *n* (%)	1,022 (47.47)
Age, years	38.91 ± 12.47
MAP, mmHg	95.35 ± 14.35
Proteinuria, g/day	0.87 (0.45–1.69)
SCr, mg/dL	1.11 ± 0.49
eGFR (ml/min/1.73 m^2^)	82.93 ± 29.28
Urine β2-MG, mg/mol Cr	56.0 (30.0–127.5)
Urine RBP, mg/mol Cr	783.0 (267.0–1774.5)
**Oxford classification**, ***n*** **(%)^a^**	
M1	197 (11.32)
E1	99 (5.69)
S1	1,221 (70.13)
T1-T2	202 (11.60)
C1-C2	704 (40.44)
**CKD stages**, ***n*** **(%)**	
1	973 (45.19)
2	637 (29.59)
3	470 (21.83)
4	73 (3.39)
**Follow-up and outcome**	
Follow-up duration, months	20.40 (9.13–43.60)
50% eGFR decline, %	126 (5.85)
ESRD, %	83 (3.86)
Composite outcome, %	140 (6.50)

*Values for continuous variables were expressed as the mean ± SD or medians [interquartile range (IQR)]; counts (percentages) were used for categorical variables. The composite outcome was defined as a 50% decrease in the eGFR or ESRD*.

*IgA, immunoglobulin A; MAP, mean arterial pressure; SCr, serum creatinine; eGFR, estimated glomerular filtration rate; β2-MG, β2-microglobulin; RBP, retinol-binding protein; M, mesangial hypercellularity; E, endocapillary hypercellularity; S, segmental glomerulosclerosis/adhesion; T, severity of tubular atrophy/interstitial fibrosis; C, presence of crescent; CKD, chronic kidney disease; ESRD, end-stage renal disease*.

a *The Oxford classification of renal pathological findings were not obtained in 412 (19.14%) cases including 33 patients with fewer than 8 glomeruli whose the Oxford classification was not performed*.

### Correlation of Urine β2-MG and Urine RBP With Clinical and Pathological Features

The Pearson's bivariate analysis found that there were significant positive correlations among the log-transformed baseline urinary β2-MG level (log β2-MG), the log-transformed baseline urinary RBP level (log RBP), and the log-transformed baseline urinary 24-h proteinuria (log proteinuria) (*r* = 0.401, *p* < 0.001 between log β2-MG and log proteinuria; *r* = 0.374, *p* < 0.001 between log RBP and log proteinuria; *r* = 0.554, *p* < 0.001 between log β2-MG and log RBP). In contrast, both the log β2-MG and the log RBP were negatively correlated with the initial eGFR (*r* = −0.360, *p* < 0.001 between log β2-MG and eGFR; *r* = −0.230, *p* < 0.001 between log RBP and eGFR). The Spearman's correlation analysis demonstrated that log β2-MG and log RBP were positively correlated with the IF/TA T score (*r* = 0.151, *p* < 0.001 between log β2-MG and the T score; *r* = 0.152, *p* < 0.001 between log RBP and the T score).

### Relationship Between Urinary β2-MG Level and Renal Outcome

Patients were stratified into 3 groups according to the urinary β2-MG tertiles and the clinical characteristics and outcomes were compared (Supplementary Table 1). The baseline urinary β2-MG levels were 20.0 mg/mol Cr (IQR, 10.0–30.0) in the first tertile, 56.0 mg/mol Cr (IQR, 45.0–74.0) in the second tertile, and 220.0 mg/mol Cr (IQR, 128.0–418.0) in the third tertile. Patients with higher baseline urinary β2-MG levels were older, had higher levels of MAP, Scr, proteinuria; had a higher percentage of IF/TA lesions in renal pathology; and lower levels of eGFR. The incidence rate of the composite renal outcome was higher in patients with higher levels of urine β2-MG (*p* < 0.001).

**Figure 2:**
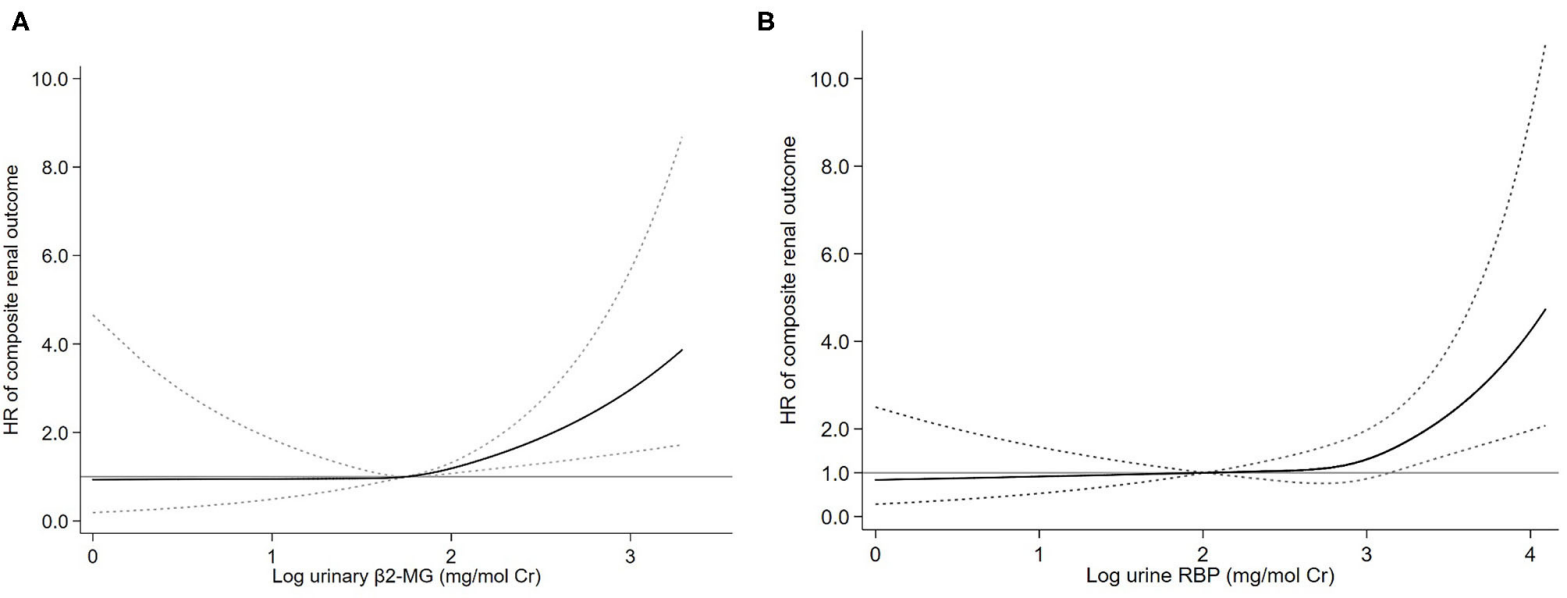
Association of log-transformed urinary β2-microglobulin (β2-MG) **(A)** and log-transformed urinary retinol-binding protein (RBP) **(B)** levels with the composite renal outcome. Three knots at the 25th, 50th, and 75th percentiles were used to model restricted cubic splines. Solid lines are multivariable adjusted hazard ratios, with dashed lines showing 95% CIs for the spline model (reference: log β2-MG 1.75 mg/mol Cr and log RBP 2.00 mg/mol Cr). Models were adjusted for age, sex, mean arterial pressure (MAP), log-transformed proteinuria, serum creatinine, and the Oxford classification (MEST-C scores).

**Figure 3:**
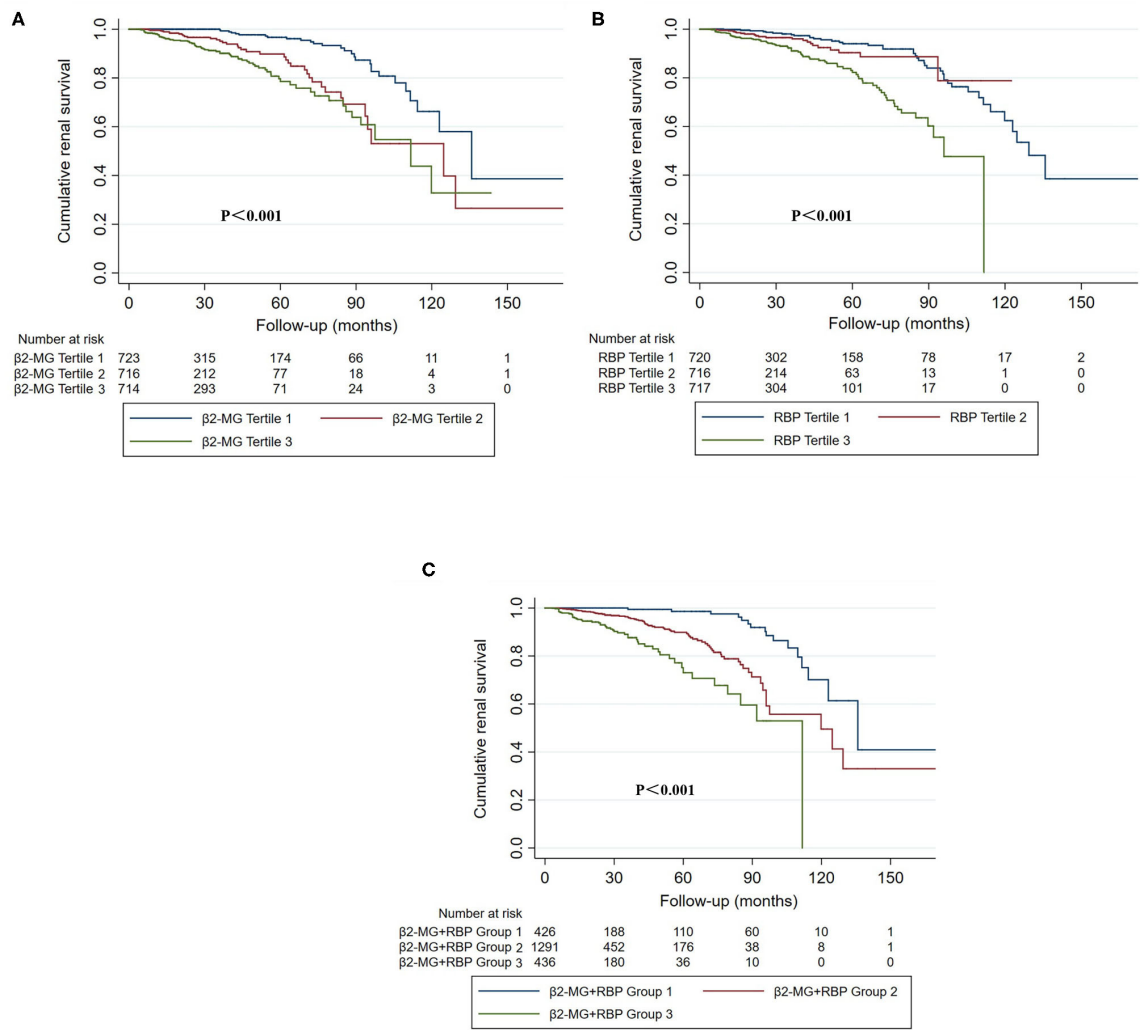
The Kaplan–Meier curves of cumulative renal survival stratified by urinary β2-MG **(A)**, urinary RBP **(B)**, tertiles and the combination of β2-MG and RBP **(C)**. **(A)** Tertile 1: the first tertile, urinary β2-MG was 20.0 mg/mol Cr [interquartile range (IQR), 10.0–30.0]; tertile 2: the second tertile, urinary β2-MG was 56.0 mg/mol Cr (IQR, 45.0–74.0); tertile 3: the third tertile, urinary β2-MG was 220.0 mg/mol Cr (IQR, 128.0–418.0). **(B)** Tertile 1: the first tertile, urinary RBP was 110.0 mg/mol Cr (IQR, 25.0–270.5); tertile 2: the second tertile, urinary RBP was 783.5 mg/mol Cr (IQR, 607.5–1014.8); tertile 3: the third tertile, urinary RBP was 2799.0 mg/mol Cr (IQR, 1774.5–4766.5). **(C)** Group 1: patients in both the first β2-MG and the first RBP tertiles; Group 3: patients in both the third β2-MG and the third RBP tertiles; Group 2: the others.

To evaluate the association between urine β2-MG and the composite renal outcome, we modeled baseline log β2-MG levels as a continuous variable using restricted cubic splines. As shown in Figure 2A, patients with higher urinary β2-MG levels had worse renal outcomes, after adjustment for age, sex, MAP, initial SCr, proteinuria, and the MEST-C scores. The Cox regression analysis indicated that baseline urine β2-MG was an independent risk factor for the composite renal disease progression outcome [+1 SD for log β2-MG, hazard ratio (HR) = 1.462, 95% CI: 1.136–1.882, *p* = 0.003] after adjusted for sex, age, MAP, proteinuria, SCr, and the MEST-C scores (Table 2). Compared to the first tertile of urine β2-MG (reference), the risk of renal disease progression increased by tertile of urinary β2-MG level: the HRs were 2.249 (95% CI: 1.102–4.588) for the second tertile, 2.921 (95% CI: 1.431–5.964) for the third tertile. As shown in Figure 3A, the renal survival deteriorated by the tertile of urinary β2-MG level. The cumulative renal survival at 3rd and 5th year in each group of patients was 99.6 and 96.7%, 95.1 and 89.8%, and 90.1 and 78.6%, respectively (log-rank test, *p* < 0.001).

**Table 2:** Association of baseline urinary β2-MG levels with the composite renal outcome.

	**Urine β2-MG, median (IQR), mg/mol Cr**	**Hazard ratio for composite outcome (95% confidence interval);** ***P*****-value**			
		**Unadjusted**	**Model 1**	**Model 2**	**Model 3**
Composite renal outcome Per 1SD Log β2-MG	56.0 (30.0–127.5)	2.023 (1.747–2.342)	1.920 (1.654–2.229)	1.427 (1.205–1.689)	1.462 (1.136–1.882)
*P*-value		<0.001	<0.001	<0.001	0.003
Urine β2-MG tertile 1	20.0 (10.0–30.0)	1 (Reference)	1 (Reference)	1 (Reference)	1 (Reference)
Urine β2-MG tertile 2 *P*-value	56.0 (45.0–74.0)	2.965 (1.816–4.844) <0.001	2.531 (1.533–4.178) <0.001	1.735 (1.040–2.893) 0.035	2.249 (1.102–4.588) 0.026
Urine β2-MG tertile 3 *P*-value	220.0 (128.0–418.0)	4.568 (2.923–7.137) <0.001	4.099 (2.614–6.427) <0.001	2.344 (1.434–3.832) 0.001	2.921 (1.431–5.964) 0.003

*The composite renal outcome was defined as a 50% decrease in the eGFR or end-stage renal disease*.

*Model 1 was adjusted for age, sex, and mean arterial pressure; sex was expressed as a dichotomous variable. Model 2 was adjusted for covariates in model 1 plus log-transformed proteinuria and SCr. Model 3 was adjusted for covariates in model 2 plus the Oxford classification (MEST-C scores)*.

*Log β2-MG, log-transformed β2-microglobulin*.

We also evaluated the relationship between baseline urine β2-MG and the composite renal outcome in patients with different renal functions. Patients were divided into two groups: eGFR ≥90 ml/min/1.73 m^2^ and eGFR <90 ml/min/1.73 m^2^. Totally, 20 (2.06%) patients in the eGFR ≥90 ml/min/1.73 m^2^ group and 120 (10.17%) patients in the eGFR <90 ml/min/1.73 m^2^ group reached the composite renal outcomes (*p* < 0.001). In the Cox regression analysis, after adjusted for sex, age, MAP, proteinuria, SCr, and the MEST-C scores, the baseline urinary β2-MG level was an independent risk factor for the composite renal outcome in patients with baseline eGFR <90 ml/min/1.73 m^2^ (+1 SD for log β2-MG, HR = 1.657, 95% CI: 1.260–2.180, *p* < 0.001). However, in patients with baseline eGFR ≥90 ml/min/1.73 m^2^, urinary β2-MG level was not associated with a poor renal outcome (+1 SD for log β2-MG, HR = 0.868, 95% CI: 0.462–1.634, *p* = 0.662).

### Relationship Between Urinary RBP Level and Renal Outcome

Clinical parameters stratified and compared according to the urinary RBP tertiles are shown in Supplementary Table 2. The baseline urinary RBP levels were 110.0 mg/mol Cr (IQR, 25.0–270.5) in the first tertile, 783.5 mg/mol Cr (IQR, 607.5–1014.8) in the second tertile, and 2799.0 mg/mol Cr (IQR, 1774.5–4766.5) in the third tertile. Patients with higher levels of baseline urine RBP were older and showed higher MAP, proteinuria, higher SCr, and lower eGFR. For renal pathological findings, IF/TA was more common in patients with higher baseline urinary RBP levels. The incidence rate of the composite renal outcome was higher in patients with higher levels of urine RBP (*p* < 0.001).

Restricted cubic splines of the association of log RBP with the composite renal outcome showed that the risk of the renal disease progression events was higher in patients with higher levels of urine RBP after adjusted for age, sex, MAP, SCr, proteinuria, and the MEST-C scores (Figure 2B). In the multivariate adjusted Cox analysis including sex, age, MAP, proteinuria, SCr, and the MEST-C scores, baseline urine RBP was an independent risk factor for the composite renal outcome (+1 SD for log RBP, HR = 1.972; 95% CI: 1.486–2.617; *p* = 0.001; Table 3). Compared to the first tertile of urine RBP (reference), the risk of the composite renal outcome increased by tertile of urinary RBP level: the HRs were 1.817 (95% CI: 0.824–4.007) for the second tertile and 2.859 (95% CI: 1.433–5.706) for the third tertile. As shown in Figure 3B, the renal survival deteriorated by the tertile of urinary RBP level. The renal survival at 3rd and 5th year in each group of patients was 97.7 and 94.0%, 96.0 and 90.3%, and 91.0 and 82.2%, respectively (log-rank test, *p* < 0.001).

**Table 3:** Association of baseline urinary RBP levels with the composite renal outcome.

	**Urine RBP, median (IQR) mg/mol Cr**	**Hazard ratio for composite outcome (95% confidence interval);** ***P*****-value**			
		**Unadjusted**	**Model 1**	**Model 2**	**Model 3**
Composite renal outcome Per 1SD Log RBP	783.0 (267.0–1774.5)	1.797 (1.513–2.135)	1.722 (1.451–2.045)	1.668 (1.403–1.984)	1.972 (1.486–2.617)
*P*-value		<0.001	<0.001	<0.001	0.001
Urine RBP tertile 1	110.0 (25.0–270.5)	1 (Reference)	1 (Reference)	1 (Reference)	1 (Reference)
Urine RBP tertile 2 *P*-value	783.5 (607.5–1014.8)	1.498 (0.869–2.582) 0.146	1.526 (0.885–2.631) 0.128	1.479 (0.853–2.564) 0.163	1.817 (0.824–4.007) 0.139
Urine RBP tertile 3 *P*-value	2799.0 (1774.5–4766.5)	3.601 (2.350–5.518) <0.001	3.305 (2.157–5.063) <0.001	2.305 (1.461–3.637) <0.001	2.859 (1.433–5.706) 0.003

*The composite renal outcome was defined as a 50% decrease in the eGFR or end-stage renal disease*.

*Model 1 was adjusted for age, sex, and mean arterial pressure; sex was expressed as a dichotomous variable. Model 2 was adjusted for covariates in model 1 plus log-transformed proteinuria and serum creatinine. Model 3 was adjusted for covariates in model 2 plus the Oxford classification (MEST-C scores)*.

*Log RBP, log-transformed retinol-binding protein*.

Furthermore, we assessed the role of baseline urine RBP in renal progression events in subgroup patients with eGFR ≥90 ml/min/1.73 m^2^ and eGFR <90 ml/min/1.73 m^2^. In the Cox regression analysis, after adjustment for sex, age, MAP, proteinuria, SCr, and the MEST-C scores, the baseline urine RBP was an independent risk factor for the composite renal outcome in patients with eGFR <90 ml/min/1.73 m^2^ (+1 SD for log RBP, HR = 1.618; 95% CI: 1.199–2.183; *p* = 0.002). However, in patients with eGFR ≥90 ml/min/1.73 m^2^, baseline urine RBP was not associated with the composite renal outcome (+1 SD for log RBP, HR = 1.625; 95% CI: 0.845–3.123; *p* = 0.146).

We also stratified all the enrolled patients into 3 groups according to the new indicator that combined the urine β2-MG with RBP. Group 1 included patients in both the first β2-MG and the first RBP tertiles, group 3 included patients in both the third β2-MG and the third RBP tertiles, and group 2 included the others. Totally, there were 426 patients in group 1, 1,291 patients in group 2, and 436 patients in group 3. Baseline clinical parameters were compared in Supplementary Table 3. Patients in group 3 had significantly higher SCr and proteinuria on biopsy and showed significantly lower baseline eGFR and worse renal outcomes. As shown in Table 4, using the 1st group as the reference, the HRs for the composite renal outcome were 3.427 (95% CI: 1.268–9.262, *p* = 0.015) in the second group and 5.596 (95% CI: 1.855–16.882, *p* = 0.002) in the third group (*p*-value for trend = 0.002) in the multivariate Cox regression analysis with adjustment of age, sex, MAP, proteinuria, SCr, and the MEST-C scores. The renal survival deteriorated by the group of the indicator that combined the urine β2-MG with RBP (Figure 3C). The renal survival at 3rd and 5th years in each group of patients was 99.4 and 98.6%, 96.1 and 89.9%, and 87.6 and 73.0%, respectively (log-rank test, *p* < 0.001).

**Table 4:** Association of the combination of baseline urine β2-MG and RBP with the composite renal outcome.

	**Hazard ratio for composite outcome (95% confidence interval);** ***P*****-value**			
	**Unadjusted**	**Model 1**	**Model 2**	**Model 3**
Urine β2-MG and RBP group 1	1 (Reference)	1 (Reference)	1 (Reference)	1 (Reference)
Urine β2-MG and RBP group 2 *P*-value	3.519 (2.019–6.134) <0.001	3.114 (1.787–5.426) <0.001	2.497 (1.385–4.503) 0.002	3.427 (1.268–9.262) 0.015
Urine β2-MG and RBP group 3 *P*-value	8.532 (4.716–15.437) <0.001	6.911 (3.822–12.493) <0.001	3.583 (1.847–6.948) <0.001	5.596 (1.855–16.882) 0.002
*P*-value for trend	<0.001	<0.001	<0.001	0.002

*The composite renal outcome was defined as a 50% decrease in the eGFR or end-stage renal disease*.

*Model 1 was adjusted for age, sex, and mean arterial pressure; sex was expressed as a dichotomous variable. Model 2 was adjusted for covariates in model 1 plus log-transformed proteinuria and SCr. Model 3 was adjusted for covariates in model 2 plus the Oxford classification (MEST-C scores)*.

*SCr, serum creatinine; eGFR, estimated glomerular filtration rate; β2-MG, β2 microglobulin; RBP, retinol-binding protein*.

## Discussion

IgA nephropathy is a highly heterogeneous disease characterized by variable clinical courses and pathologic findings ranging from asymptomatic microhematuria with mild mesangial proliferation to rapidly progressive glomerulonephritis with extensive crescents formation (22). Several clinical and pathologic parameters have been identified in associations with IgAN progression including blood pressure, proteinuria, baseline renal function, and the Oxford classification of the MEST-C scores (5–11, 23, 24). However, the individual outcomes of IgAN remain difficult to predict. Tubulointerstitial damage is common in IgAN. A study of 609 Chinese patients with IgAN found that nearly 38.3% of the included patients had moderate or severe tubulointerstitial lesions at the time of renal biopsy (15). *In-vitro* studies have suggested that a mesangial-podocytic-tubular cross-talk with mediators released from the mesangium along with the filtered proteins from the impaired podocyte, contributes to the pathogenesis of tubulointerstitial damage in IgAN (13, 25). Clinically, a subgroup of IgAN with proximal tubular epithelial cells and tubulointerstitial damage often is associated with rapid progression to ESRD (14, 25). Thus, detection of tubulointerstitial lesions in early stage, especially by non-invasive biomarkers, is of clinical significance. Tubulointerstitial damage leads to the dysfunctional proximal reabsorption of physiologically filtered low-molecular-weight proteins and increases urine concentrations of these substances. Urine β2-MG and urine RBP are thought as typical markers for estimation of tubulointerstitial malfunction (19, 26, 27). This study suggested that baseline urine β2-MG and RBP can be used as additional variables in prediction of progression in IgAN.

β2-microglobulin is a low-molecular-weight protein present in low levels in normal human plasma and body fluid. Elevated serum β2-MG levels may result from decreased glomerular filtration function or increased filtration loads such as chronic inflammatory diseases and hematological malignancies (16, 28). Though serum levels of β2-MG were not measured in this study, patients accompanied by conditions that may result in highly increased serum β-MG were excluded. β2-MG is excreted in large amounts in the urine of patients with tubulointerstitial diseases and it is a sensitive marker for early detection of tubular dysfunction (29, 30). In 1986, Portman et al. (18) diagnosed proximal tubular dysfunction by testing urine β2-MG and found an association between higher urinary levels of β2-MG and progression to ESRD in children. Previous studies found that in patients with IgAN, urine β2-MG was positively correlated with SCr and the severity of tubulointerstitial lesions (19, 31). However, few studies investigated the relationship between urine β2-MG and renal disease progression of IgAN and the results remain controversial due to the limited number of patients included (31, 32). Therefore, larger studies with more participants are needed to identify urine β2-MG as a marker of the renal disease progression events in clinical practice. This study found that elevated urine level of β2-MG was independently associated with increased risk of renal disease progression in IgAN. Among cases in this study, participants with the highest vs. the lowest tertile demonstrated a 2.921-fold greater risk of renal progression.

Retinol-binding protein is also a low-molecular mass protein and increased urine RBP excretion also indicates tubular dysfunction (33, 34). Previous studies demonstrated that urine RBP was as sensitive as β2-MG in screening renal tubular function and was a marker of the extent of IF/TA in chronic kidney disease (20, 35, 36). Numerous studies have reported an increased urinary excretion of RBP in diabetic nephropathy and idiopathic membranous nephropathy and recognized the correlations of urine RBP with tubulointerstitial injury and disease progression (36–39). However, the role of urine RBP as a marker of tubulointerstitial injury and renal disease progression in IgAN has not been elucidated. This study found that an increased level of urine RBP was independently associated with the renal disease progression events. Patients with the highest vs. the lowest tertile showed a 2.859-fold greater risk of renal progression.

Consistent with previous studies, this study showed that both the baseline urinary β2-MG and RBP levels were significantly correlated with traditional risk factors of renal disease progression, such as proteinuria, renal dysfunction, and the IF/TA T score in patients with IgAN. Notably, we also found that baseline urine β2-MG and RBP were strongly associated with renal disease progression events in IgAN even after adjustment for sex, age, MAP, SCr, proteinuria, and the Oxford classification of the MEST-C scores, especially in those with eGFR <90 ml/min/1.73 m^2^. However, only 20 patients in eGFR ≥90 ml/min/1.73 m^2^ group reached the composite renal outcomes and the differences may result from the relatively short follow-up period for these patients, with fewer endpoints observed. Further studies with extensive follow-up duration are needed for patients with baseline eGFR ≥90 ml/min/1.73 m^2^.

This is a large sample size study with a relatively large number of renal disease progression events. To the best of our knowledge, this is the first study to evaluate the role of urine RBP in renal disease progression in IgAN and also the largest study to evaluate the role of urine β2-MG in IgAN. However, there are some limitations of this study. First, it was a retrospective cohort study performed in a single center and the follow-up time was relatively short. Also, the baseline Oxford classification of renal biopsy was not obtained in 19.14% of all the participants. Second, information about 24-h urinary β2-MG and RBP excretion was not available, so we used a spot urine sample for testing urinary β2-MG and RBP and corrected the results by urine creatinine. Third, due to data limitations, treatments such as the use of renin-angiotensin-aldosterone system inhibitors (RAASi), steroids, and other immunosuppressants were not examined during follow-up and our analyses were not adjusted for these factors. In addition, urine β2-MG and RBP were highly skewed to the right in this cohort of patients; therefore, log-transformation and tertiles of urinary β2-MG and RBP levels were used in the Cox proportional hazards models. Finally, we recognize that our results may not be applicable to all the populations, as patients with IgAN in our cohort showed milder pathological lesions than other IgAN cohorts (15); though we have included the Oxford classification of the MEST-C scores as confounders in the multivariate adjusted Cox models, further studies are needed to confirm the findings.

In conclusion, in this study, we demonstrated that baseline levels of urine β2-MG and RBP correlated with proteinuria, eGFR, and the Oxford classification of IF/TA T score. Higher levels of urine β2-MG and RBP were associated with renal disease progression in patients with IgAN. Our findings suggest that baseline levels of urine β2-MG and RBP may have prognostic utility in IgAN.

## Data Availability Statement

The original contributions presented in the study are included in the article/Supplementary Material, further inquiries can be directed to the corresponding author/s.

## Author Contributions

JCheng and XS conceived the study. XS, GY, XL, and HL performed data collection and analyses. XS prepared the first draft of the manuscript. JChen and JCheng reviewed and provided revisions and comments to the manuscript. All the authors contributed to the article and approved the submitted version of the manuscript.

## Conflict of Interest

The authors declare that the research was conducted in the absence of any commercial or financial relationships that could be construed as a potential conflict of interest.

## Publisher's Note

All claims expressed in this article are solely those of the authors and do not necessarily represent those of their affiliated organizations, or those of the publisher, the editors and the reviewers. Any product that may be evaluated in this article, or claim that may be made by its manufacturer, is not guaranteed or endorsed by the publisher.
