# Supplementary material for: Urine β2-Microglobulin and Retinol-Binding Protein and Renal Disease Progression in IgA Nephropathy
Source: Front Med (Lausanne). 2021 Dec 22;8:792782. doi: 10.3389/fmed.2021.792782 (PMC8727481; doi:10.3389/fmed.2021.792782)
Supplement: Supplementary file 1 [file Data_Sheet_1.docx]

**Table S1. Clinical characteristics and outcomes for patients stratified by urinary β2-MG tertiles**

|  | Urinary β2-MG tertiles, median (IQR), mg/mol Cr | | | P-value |
| --- | --- | --- | --- | --- |
|  | Tertile 1  20.0 (10.0-30.0)  N=723 | Tertile 2  56.0 (45.0-74.0)  N=716 | Tertile 3  220.0 (128.0-418.0)  N=714 |  |
| Male sex, n (%) | 380 (52.6) | 308 (43.0) | 334 (46.8) | 0.001 |
| Age, years | 35.72±11.00 | 39.75±12.06 | 41.30±13.58 | ＜0.001 |
| MAP, mmHg | 93.64±13.91 | 94.60±14.07 | 97.82±14.74 | ＜0.001 |
| Proteinuria, g/day | 0.56 (0.29-1.10) | 0.81 (0.47-1.46) | 1.37 (0.79-2.68) | ＜0.001 |
| SCr, mg/dL | 0.96±0.37 | 1.07±0.48 | 1.28±0.57 | ＜0.001 |
| eGFR, ml/min/1.73m^2^ | 94.68±25.01 | 83.05±27.43 | 70.92±30.25 | ＜0.001 |
| Oxford classification of T1-T2, n (%) | 29 (5.2) | 71 (11.3) | 102 (18.4) | ＜0.001 |
| Composite outcome, n (%) | 28 (3.9) | 40 (5.6) | 72 (10.1) | ＜0.001 |

Abbreviations: MAP, mean arterial pressure; SCr, serum creatinine; eGFR, estimate glomerular filtration rate; β2-MG, β2-microglobulin

**Table S2. Clinical characteristics and outcomes for patients stratified by urinary RBP tertiles**

|  | Urinary RBP tertiles, median (IQR), mg/mol Cr | | | P-value |
| --- | --- | --- | --- | --- |
|  | Tertile 1  110.0 (25.0-270.5)  N=720 | Tertile 2  783.5 (607.5-1014.8)  N=716 | Tertile 3  2799.0 (1774.5-4766.5)  N=717 |  |
| Male sex, n (%) | 382 (53.1) | 340 (47.5) | 300 (41.8) | ＜0.001 |
| Age, years | 38.10±12.24 | 38.09±11.60 | 40.55±13.36 | ＜0.001 |
| MAP, mmHg | 93.65±13.91 | 95.12±14.03 | 97.27±14.87 | ＜0.001 |
| Proteinuria, g/day | 0.50 (0.26-1.14) | 0.76 (0.47-1.22) | 1.51 (0.89-2.58) | ＜0.001 |
| SCr, mg/dL | 1.02±0.40 | 1.10±0.48 | 1.20±0.57 | ＜0.001 |
| eGFR, ml/min/1.73m^2^ | 89.36±27.14 | 83.57±28.83 | 75.84±30.25 | ＜0.001 |
| Oxford classification of T1-T2, n (%) | 39 (7.5) | 61 (9.6) | 102 (17.6) | ＜0.001 |
| Composite outcome, n (%) | 43 (6.0) | 23 (3.2) | 74 (10.3) | ＜0.001 |

Abbreviations: MAP, mean arterial pressure; SCr, serum creatinine; eGFR, estimate glomerular filtration rate; RBP, retinol binding protein

**Table S3. Clinical characteristics and outcomes for patients stratified by combination of urine β2-MG and RBP tertiles**

|  | Combination of Urine β2-MG and RBP | | | P-value |
| --- | --- | --- | --- | --- |
|  | Group 1  N=426 | Group 2  N=1291 | Group 3  N=436 |  |
| Male sex ,n (%) | 234 (54.9) | 602 (46.6) | 186 (42.7) | ＜0.001 |
| Age, years | 35.77±11.36 | 39.11±12.09 | 41.40±13.91 | ＜0.001 |
| MAP, mmHg | 93.29±14.14 | 95.00±13.99 | 98.37±15.15 | ＜0.001 |
| Proteinuria, g/day | 0.42 (0.23-0.99) | 0.81 (0.47-1.44) | 1.78 (1.08-3.14) | ＜0.001 |
| SCr, mg/dL | 0.96±0.37 | 1.09±0.47 | 1.31±0.60 | ＜0.001 |
| eGFR, ml/min/1.73m^2^ | 95.89±25.29 | 83.29±28.12 | 69.21±30.30 | ＜0.001 |
| Oxford classification of T1-T2, n (%) | 14 (4.9) | 119 (10.6) | 69 (21.0) | ＜0.001 |
| Composite outcome, n (%) | 16 (3.8) | 74 (5.7) | 50 (11.5) | ＜0.001 |

Abbreviations: MAP, mean arterial pressure; SCr, serum creatinine; eGFR, estimate glomerular filtration rate; β2-MG, β2-microglobulin; RBP, retinol binding protein.
